# A Modular and Practical Synthesis of Zwitterionic Hydrogels through Sequential Amine-Epoxy “Click” Chemistry and N-Alkylation Reaction

**DOI:** 10.3390/polym11091491

**Published:** 2019-09-12

**Authors:** Junki Oh, Kevin Injoe Jung, Hyun Wook Jung, Anzar Khan

**Affiliations:** Department of Chemical and Biological Engineering, Korea University, Seoul 02841, Korea; ojk0911@naver.com (J.O.); ij@grtrkr.korea.ac.kr (K.I.J.); hwjung@grtrkr.korea.ac.kr (H.W.J.)

**Keywords:** amine-epoxy reaction, “click” chemistry, poly(β-hydroxyl amine)s, hydrogel synthesis, sequential reactions, “click” hydrogels, hydrogel patterning

## Abstract

In this work, the amine-epoxy “click” reaction is shown to be a valuable general tool in the synthesis of reactive hydrogels. The practicality of this reaction arises due to its catalyst-free nature, its operation in water, and commercial availability of a large variety of amine and epoxide molecules that can serve as hydrophilic network precursors. Therefore, hydrogels can be prepared in a modular fashion through a simple mixing of the precursors in water and used as produced (without requiring any post-synthesis purification step). The gelation behavior and final hydrogel properties depend upon the molecular weight of the precursors and can be changed as per the requirement. A post-synthesis modification through alkylation at the nitrogen atom of the newly formed β-hydroxyl amine linkages allows for functionalizing the hydrogels. For example, ring-opening reaction of cyclic sulfonic ester gives rise to surfaces with a zwitterionic character. Finally, the established gelation chemistry can be combined with soft lithography techniques such as micromolding in capillaries (MIMIC) to obtain hydrogel microstructures.

## 1. Introduction

**Scheme 1:**
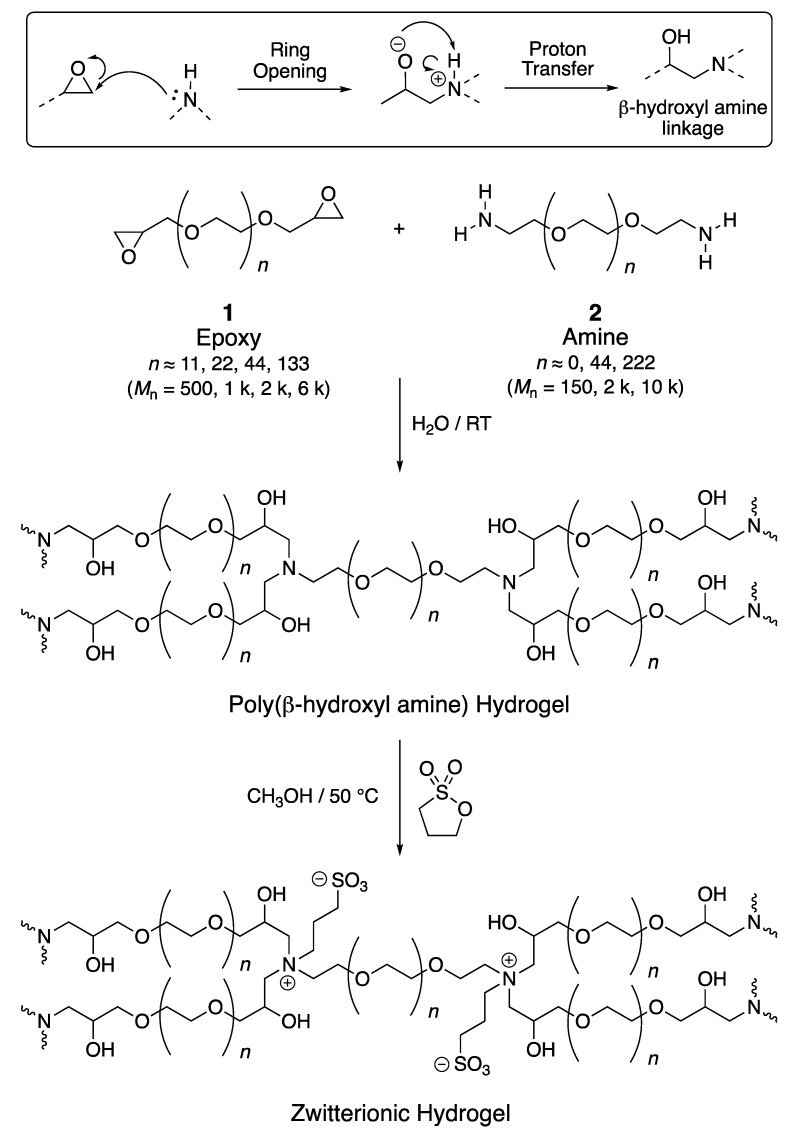
Catalyst-free ring-opening amine-epoxy reaction to prepare hydrogels through use of poly(ethylene glycol)-based precursors and subsequent gel functionalization through alkylation of the nitrogen atoms to access zwitterionic materials. The wavy lines represent extension of the cross-linked structure. It is likely that the hydrogels contain many structural defects arising from un-reacted functional groups. Therefore, the structures drawn are ideal and should be treated with caution.

The strained three-membered ring of an epoxide group can be opened by a variety of nucleophiles [1,2]. For example, carboxylic acid, phenol, alcohol, and thiol-based nucleophiles can give efficient access to polyesters, polyethers, and polythioethers when treated with appropriately designed epoxy monomers in the presence of a base catalyst [3,4,5,6,7,8,9,10,11,12,13,14,15]. The fifth general class of organic nucleophiles is represented by alkyl amines. Due to their inherent nucleophilicity, unlike the aforementioned processes, they do not require a catalyst for the ring-opening reaction to commence (Scheme 1). Furthermore, water is discussed as an ideal medium for such reactions as it can satisfy a range of hydrogen bonding situations arising during an epoxy ring-opening reaction [16]. The amine-epoxy reaction is also efficient and described by Sharpless as a potential “click” reaction [16]. Its orthogonality with other “clickable” groups such as acetylene, ene, and azides also bodes-well for the preparation of multiply-functionalized soft materials [17,18,19,20,21,22,23,24]. Finally, a large number of amines and epoxides are commercially available. Therefore, the amine-epoxy reaction is of considerable interest to a polymer chemist due to its simplicity, efficiency, chemoselectivity, practicality, and the potential to be carried out under environmentally friendly conditions. One particular avenue is the synthesis of hydrophilic polymer networks [25,26,27,28,29,30,31,32,33,34,35,36,37,38,39,40]. Despite the beneficial attributes of the amine-epoxy reaction; its application for the synthesis of hydrogels remains restricted to a handful of specific examples [41,42,43,44,45,46,47,48,49,50,51]. Furthermore, so far none of these studies employ the tertiary amine sites for direct functionalization of the generated β-hydroxyl amine scaffolds. Since quaternary ammonium groups are vital in the design of antibiofouling materials, the potential of transforming the amine-epoxy hydrogels in one post-gelation step into a zwitterionic structure would, therefore, represent a considerable advance in the chemistry and applications of such bio-relevant materials. 

Towards this end, in this study, we establish modularity and practicality of the amine-epoxy reaction for the synthesis of hydrogels with tunable properties. After hydrogel synthesis, the nitrogen atoms of the hydrophilic network are employed in installation of a functional group through alkylation reaction. Such a post-gelation modification reaction transforms the chemically neutral network to an ionic material through quaternization of the amine groups. Finally, the concept of forming the gel via the amine-epoxy “click” reaction is combined with the concept of micro-fabrication to obtained hydrogel micro-patterns.

## 2. Materials and Methods 

### 2.1. Experimental Details

Poly(ethylene glycol) diglycidyl ether **1** (*M*_n_ = 500, 1000, 2000, 6000 g/mol), 2,2′-(ethylenedioxy)bis(ethylamine), propylene oxide, ethanolamine, 1,3-propane sultone, and albumin-fluorescein isothiocyanate conjugate (BSA-FITC, product no. A9771) were purchased from Sigma Aldrich (St. Loius, MA, USA). PEG-Amine **2** (*M*_n_ = 2000, 10,000 g/mol) was purchased from Laysan Bio. Infrared (IR) analyses were carried out using an attenuated total reflection (ATR)-Fourier transform infrared (FTIR) spectrometer (PerkinElmer Spectrum Two FT-IR Spectrometer). The surface and inside of Hydrogels were characterized by a field emission-scanning electron microscope (FE-SEM, Hitach S-4800, Hitachi High-Technologies, Tokyo, Japan) operated at 15 kV. X-ray photoelectron spectroscopy (XPS) analysis was carried out with AXIS SUPRA (Kratos, UK) using Automated Monochromatic X-ray source. Narrow scan analyses were carried out with a pass energy of 20 eV and a step size of 0.1 eV. NMR spectra were recorded on a Varian NMR system 500 MHz spectrometer (VNMRS500) using D_2_O as a solvent.

**5**: Propylene oxide (**3**) (1.74 g, 30 mmol) and ethanolamine (**4**) (0.61 g, 10 mmol) were dissolved in 5 mL of DI water. The reaction mixture was stirred at room temperature overnight and freeze-dried without further purification. Afterwards, the sample was analyzed by ^1^H NMR. ^1^H NMR (500 MHz, Deuterium Oxide) δ 4.03 (m, 2H), 3.87–3.69 (m, 2H), 3.14–2.64 (m, 6H), 1.18 (m, 6H). In an alternative procedure, the reaction was carried out for 3 h at 70 °C. The crude product was identified to be **5** through ^1^H-NMR spectroscopy. 

**9**: **5** (0.354 g, 2 mmol) and 1,3-propanesultone (**8**) (0.488 g, 4 mmol) were dissolved in 5 mL of methanol. The reaction mixture was stirred at 50 °C for 48 h. After the reaction, mixture was precipitated into cold diethyl ether. The precipitate was separated by centrifugation and the collected liquid dried under high vacuum conditions. ^1^H NMR (500 MHz, Deuterium Oxide) δ 4.30–4.17 (m, 2H), 3.9 (t, 2H), 3.58 (t, 2H), 3.55–3.18 (m, 6H), 2.9 (t, 2H), 2.04–1.95 (m, 2H), 1.2 (d, 6H).

### 2.2. Gel Functionalization

The dried hydrogel and 1,3-propanesultone (1 M) were added in methanol. The reaction mixture was stirred at 50 °C for 48 h. After the reaction, sulfonated hydrogel was dialyzed in methanol for 24 h and deionized water for 24 h.

### 2.3. Preparation of Hydrogels

A typical procedure (Example = Entry 1, Table 1): Poly(ethylene glycol) diglycidyl ether (*M*_n_ = 500 g/mol, 1 g, 2 mmol) was added to 1 mL of deionized (DI) water and the mixture was stirred until it became a clear solution. Subsequently, 2,2′-(ethylenedioxy)bis(ethylamine) (0.15 g, 1 mmol) was added to the solution and vigorously stirred for 5 min. The gelation process was monitored by tube-inversion method and the gel was washed with water and freeze-dried before any further use. The amount, temperature, gelation time etc. details can be seen in other entries of Table 1 and Table 2. In general, for longer precursors, a longer stirring time was necessary to obtain a homogenous aqueous solution. For gelation at 70 °C, the samples were kept in a pre-heated oven. In all cases, the weight of the dried hydrogels was equal to the combined weight of the precursors. 

### 2.4. Rheological Characterization

The sol-gel transition of hydrogels was determined by a rotational rheometer (AR2000, TA Instruments, New Castle, DE, USA). The polymer aqueous solution was placed between parallel plates of 40 mm diameter and a gap of 0.5 mm at room temperature. Elastic (G′) and viscous (G″) moduli were investigated in the small amplitude oscillatory shear (SAOS) mode with a frequency of 1 Hz and 1 % strain amplitude under linear viscoelasticity conditions.

### 2.5. Mechanical Tests

The measurement of mechanical properties of the hydrogels was conducted on a universal testing machine using an MTDI UT-005F Series (MINOS-005) at a rate of 1 mm/min and at room temperature. The hydrogels were prepared and immersed in DI water until they became fully swollen. The diameter and height of the dry hydrogels were kept constant at 12 and 3 mm, respectively. The swollen hydrogels were taken out of the water just before the measurement. 

### 2.6. Swelling Studies

Hydrogels were freeze-dried. The initial mass of dried hydrogels was measured and then they were immersed in distilled water until they reached the equilibrium state. Swollen hydrogels were weighed, and swelling was calculated according to the equation, Water Uptake (%) = ((mass of swollen hydrogel-mass of dried hydrogel)/mass of dried hydrogel) × 100.

### 2.7. Thermogravimetric Analysis 

The decomposition profile of the hydrogel was analyzed with a TA Instruments (Utah, New Castle, DE, USA) Q50. 8 mg of a hydrogel sample was placed in a platinum sample pan and heated from 25 to 700 °C under a nitrogen atmosphere at a heating rate of 10 °C/min, and the weight loss was recorded as a function of temperature.

### 2.8. Protein Adsorption on the Hydrogel

BSA-FITC was dissolved in phosphate buffered saline (PBS, 10 mM, pH 7.4) to a protein concentration of 2 mg/mL. Fresh hydrogel and functionalized hydrogel are immersed with the dissolved protein solution for 24 h at 37 °C. The solution was wrapped with aluminum foil to provide a dark environment. The hydrogels were then washed twice with PBS and once with deionized water to remove weakly adhered BSA-FITC. The hydrogels were then viewed under an Olympus BX53 microscope equipped with a 470 nm excitation filter and a 540 nm emission filter for green fluorescence. For quantitative analysis, NIH ImageJ software (http://imagej.nih.gov/ij/, Madison, WI, USA) was used to analyze the images obtained from fluorescence microscopy.

### 2.9. MIMIC Lithography

Poly(ethylene glycol) diglycidyl ether (*M*_n_ = 2000 g/mol, 0.2 g, 0.1 mmol) and NH_2_–PEG–NH_2_ (*M*_n_ = 2000 g/mol, 0.4 g, 0.05 mmol) were added in 0.6 g DI water and stirred for 1 h at room temperature. After stirring, the mixture was impregnated between the silicon master and the silicon wafer and left overnight in 70 °C before peeling off from the silicon master with tweezers.

## 3. Results and Discussion

**Figure 1:**
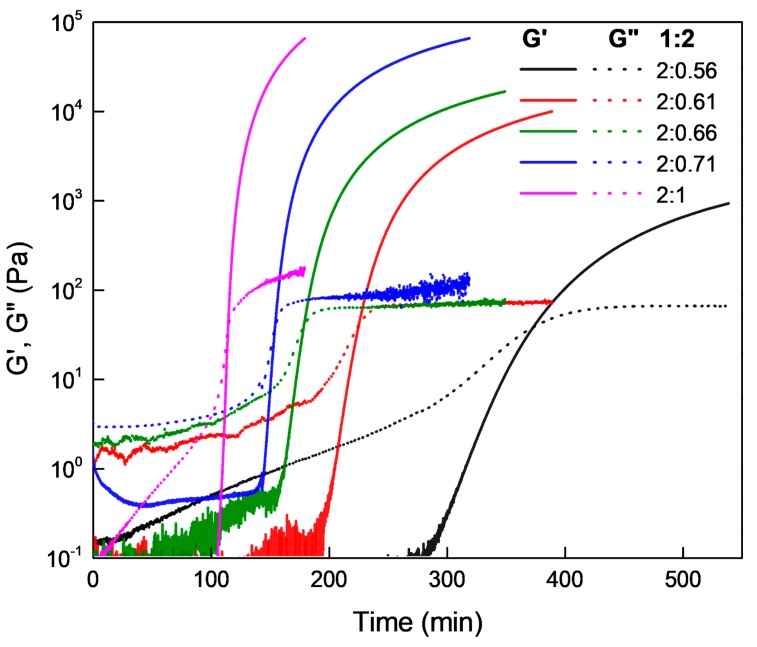
Real-time rheological study involving precursors 1 (*M*_n_ = 500 g/mol) and 2 (*M*_n_ = 150 g/mol) with varying molar ratios.

In amine-epoxy reaction, a primary amine reacts twice to the glycidyl units. Therefore, diglycidyl ether **1** and di-amine **2** were chosen as precursors to afford a non-linear structure (Scheme 1). The hydrophilicity in **1** and **2** comes from the presence of the ethylene-oxide units [52,53,54]. Initially, low molecular weight compounds (*M*_n_ = 500 and 150 g/mol) were employed to study the gelation reaction (Table 2). To accomplish this, rotational rheometer was used for comparing the elastic (G′) and viscous (G″) moduli as a function of reaction time. Before gelation, the value of G″ is greater than G′ and indicates that the sample has a liquid-like behavior. With the progress of reaction, G′ value dramatically increases and eventually exceeds G″ value. This indicates that the liquid precursor solution transformed into a solid-like gel state. In this data, the crossover of viscous and elastic moduli indicates the exact reaction time at which the precursor solution becomes a gel. With this study, it became clear that gelation occurred only above the threshold molar ratio of 2:0.5. An increase in the amount of amine accelerated the gelation reaction and fast and full gelation required use of a 2:1 molar ratio between **1** and **2** (Figure 1). The gelation was done in water and at room temperature. 

**Figure 2:**
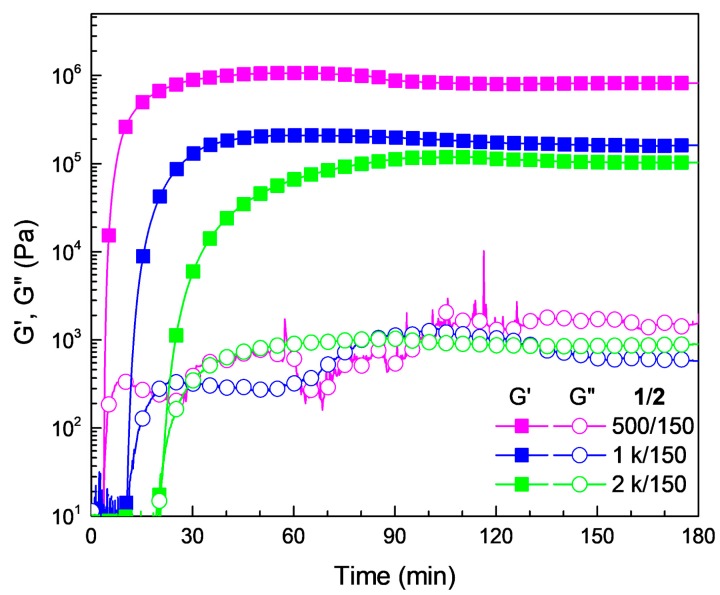
Real-time rheological data with varying length of the precursors 1 and 2 (Table 2 entries 1, 4, and 7).

After having studied the effect of molar ratios on gelation, relatively higher molecular weight epoxy precursors with *M*_n_ of 1, 2, and 6 kDa were used (Table 1 entries 4, 7, and 10. Figure 2 and Figure S1 in Supplementary Materials). However, due to gelation time in hours, the gelation temperature was changed from room temperature to 70 °C. Under these conditions, the gelation time could be reduced to a few minutes. The largest molecular weight epoxy precursor (6 kDa) still failed to form a robust gel (Figure S1 in Supplementary Materials).

The next variable was to use higher molecular weight amines (*M*_n_ = 2 and 10 kDa) in combination with the low molecular weight epoxy precursor (*M*_n_ = 500 g/mol) (Table 2 entries 2–3). The gelation happened successfully in both cases. However, the gelation time was considerably longer for the longer (10 kDa) amine precursor (Figure S1 in Supplementary Materials). 

Finally, the high molecular weight epoxy and amine precursors were combined (Table 2 entries 5, 6, 8, 9, 11, and 12). For combinations involving 1 and 2 kDa precursors, the gelation occurred in a matter of minutes. For systems involving 10 kDa amine precursor, a gelation time of a few hours was required. Once again 6 kDa epoxy precursor failed to give robust gels.

**Figure 3:**
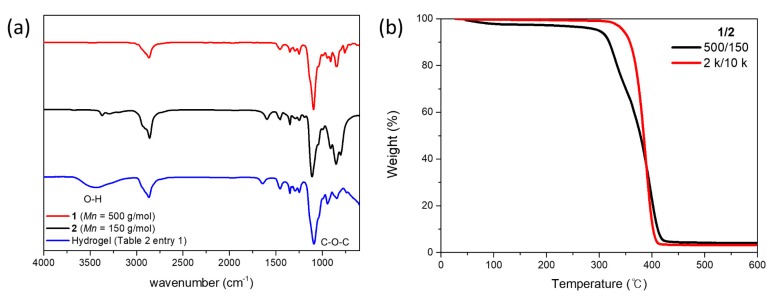
(**a**) IR Infrared spectra of precursors 1 and 2 and hydrogel (Table 1 entry 1). (**b**) Thermogravimetric analysis of hydrogels (Table 1 entries 1 and 9).

In IR spectroscopy, the hydroxyl groups generated through the ring-opening reaction of the epoxide groups could be located as a broad signal at 3500 cm^−1^ (Figure 3a). The ether C–O stretch from the ethylene oxide segments could be observed as an intense signal at 1100 cm^−1^. Thermogravimetric analyses suggested that the hydrogels were thermally stable up to 300 °C (Figure 3b). 

**Figure 4:**
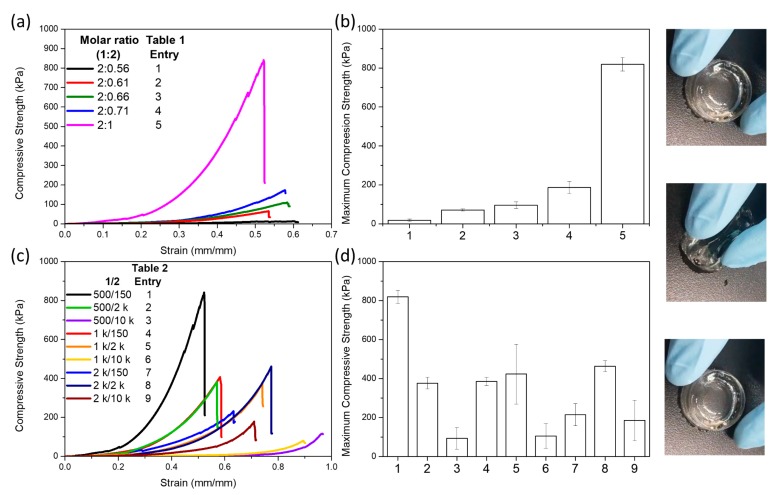
Compressive strength test for the hydrogels. (**a**,**b**) The top shows data for entries 1–5 in Table 2. (**c**,**d**) The bottom shows data for entries 1–9 in Table 1. The digital picture shows a sample made from 2 kDa precursors (Table 1 entry 8).

The mechanical properties of the hydrogels were studied in their fully swollen state through compressive tests (Figure 4). These tests were carried out six times to ensure reliability of the acquired data. This study showed that hydrogels made from shorter precursors were stiffer, presumably due to a higher number of networking points per unit volume. The larger precursor led to softer materials. A good balance between flexibility and stiffness was achieved when medium sized precursors were employed. For example, gels made from a 1 and 2 kDa precursors showed compressive strength in the range of 400–500 kPa and material extension in the range of 56–77%. 

**Figure 5:**
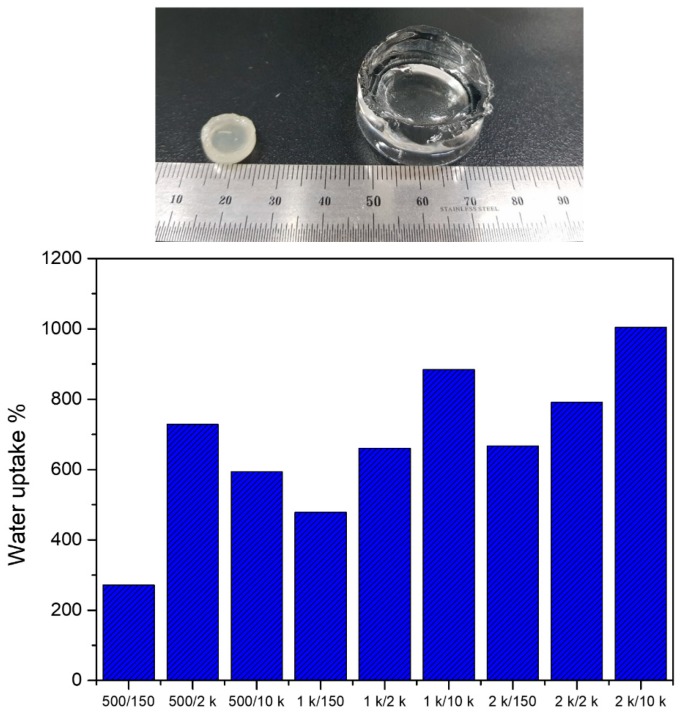
Water uptake capacity of the hydrogels. Digital picture shows the hydrogel (Table 1 entry 9) in its dry (**left**) and swollen states (**right**).

Swelling behavior of the hydrogels was then examined (Figure 5). For this, the materials were freeze-dried and then swollen in water. The water uptake capacity was measured by gravimetric analysis until an equilibrium swelling state was reached. For hydrogels made from short precursors (*M*_n_ = 150 and 500 g/mol), the water uptake capacities were relatively low (250–750%). Hydrogels from larger precursors could uptake larger amounts of water. The highest capacity of 1000% was observed for materials having the largest precursor (10 kDa).

**Figure 6:**
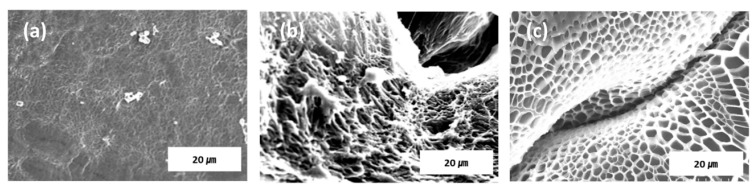
Scanning electron micrographs of the hydrogels from entries 7 (**a**), 8 (**b**), and 9 (**c**) of Table 2.

Finally, the internal morphology of the hydrogels was observed with the help of scanning electron microscopy (SEM) in their freeze-dried state (Figure 6 and Figure S2 in Supplementary Materials). Hydrogels made from short precursors were dense and a microporous structure could not be seen (Figure 6a). This is likely due to a high crosslinking density of the materials. Hydrogels made from longer precursors, however, showed microporous internal structure. When both precursors were polymeric, we were able to observe a uniform and homogenous porosity (Figure 6c).

**Scheme 2:**
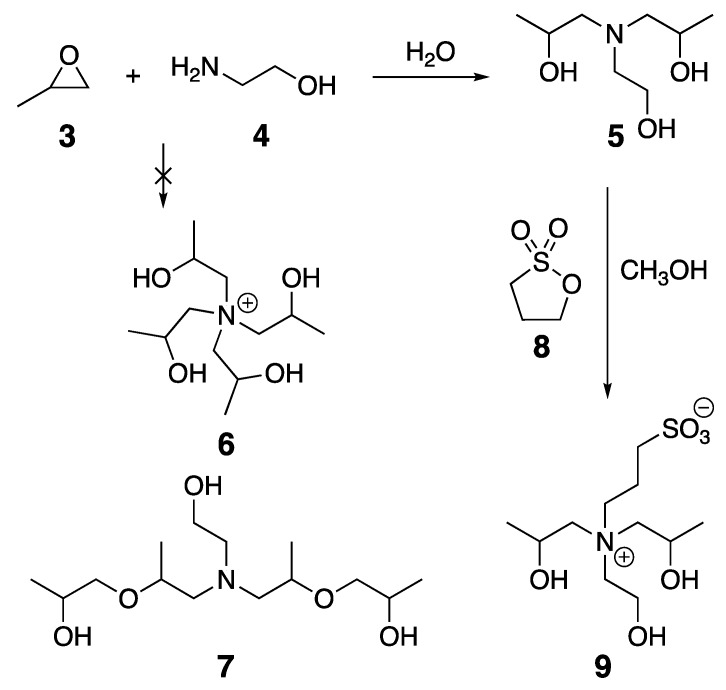
A model compound study to investigate structural defect formation during gelation and optimum conditions for quaternization of the nitrogen atoms after gelation.

Having practical access to the hydrogels, their functionalization through alkylation at the nitrogen atom was considered. However, a small molecular model compound study was first undertaken to establish the optimum conditions for the functionalization. Unlike hydrogels, the small molecule-based reactions can be unambiguously characterized through solution-based techniques such as ^1^H-NMR spectroscopy. For this, epoxide **3** and amine **4** were initially used to obtain the fundamental β-hydroxyl amine motif **5** (Scheme 2). This reaction is of importance as well because it indicates whether the amine-epoxy reaction produces the targeted structure only or side-products such as the over-alkylation at the nitrogen atom (**6**) and/or the hydroxyl group (**7**) compromises the structural integrity of the material. Area integration analysis in ^1^H-NMR spectroscopy indicates that only two new alkyl amine linkages formed upon reaction with glycidyl methyl ether at room temperature for 12 h or at 70 °C for 3 h (Figure S3 in Supplementary Materials). The over-alkylation would have resulted in mismatching of the integration ratio between the protons located adjacent to the secondary hydroxyl groups and the primary hydroxyl group. Absence of over-alkylation in the present system stems from the fact that the hydroxyl groups pulls the electron density away from the nitrogen atom thus making it less nucleophilic and less prone to further alkylation at room temperature or for a short period of time (3 h) at elevated temperature (70 °C) (the gel forming conditions).

**Figure 7:**
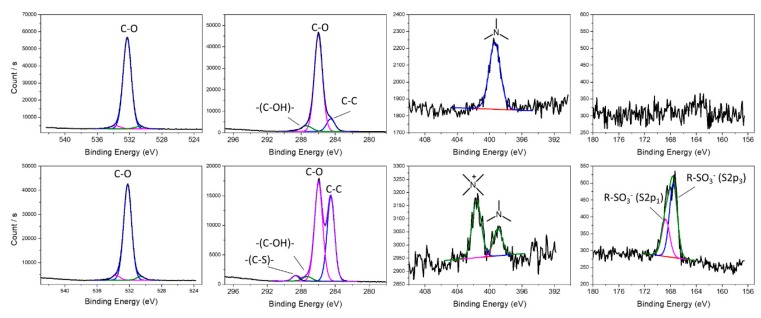
X-ray photoelectron spectra of the hydrogel before (**top**) and after alkylation (**bottom**).

The cationization of the nitrogen atom was then investigated through ring-opening reaction of cyclic sulfonic ester **8**. As can be expected from the aforementioned discussion, such alkylation reaction failed under gelation conditions and occurred only when a large excess of the alkylating agent was stirred at elevated temperature (50 °C) for a long period of time (≥48 h). The product (**9**) displayed an expected 1:1 integration ratio between the two terminal methyl groups (labeled “a” in Figure S4 in Supplementary Materials) and the protons of the alkyl chain from the ring-opening reaction (labeled e/f/g in Figure S4 in Supplementary Materials). The optimized conditions were then applied on the best (2 kDa/2 kDa) hydrogel system. X-ray photoelectron spectroscopy indicated that the conversion of the tertiary amine to the quaternary amine was 68.5 % (Figure 7). Expected signal from the sulfur atom of the sulfonate was also observed at 167 eV. Due to an increase in the carbon content of the material upon sulfonation, the signal belonging to the C–C bond at 184 eV also increased in intensity. Overall, these results indicated a successful alkylation of the nitrogen atoms through ring-opening reaction and formation of a sulfonate/ammonium-based zwitterionic motif. In light of this, a preliminary antibiofouling study was carried out. In this study, the gels were exposed to fluorescein labeled albumin for 24 h. The exposed gels were then examined with the help of fluorescence microscopy by exciting the fluorescein dye at 470 nm and reading out of its emission signal at 540 nm. This study indicated that the fluorescence intensity in the zwitterionic gel decreased to approximately 1/3 of that measured in the un-functionalized gels (Figure S5 in Supplementary Materials). 

**Figure 8:**
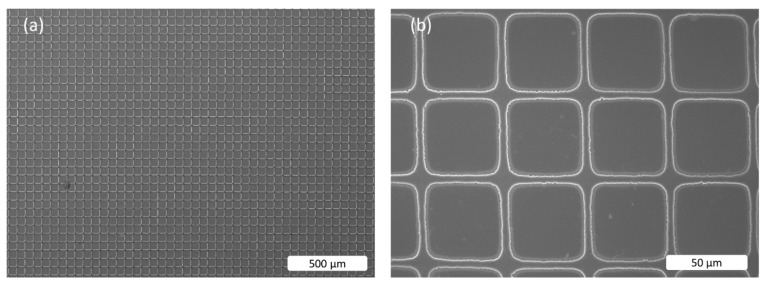
Scanning electron micrographs (SEM) of grid (line width of ca. 10 μm, height ca. 1 μm) pattern at different lower (**a**) and higher (**b**) magnifications produced by micromolding in capillaries technique.

The final goal of this work was to establish whether soft lithography techniques could be used to pattern the hydrogels. For this, initially, utility of micromolding in capillaries (MIMIC) was examined in the present context [55]. In this method established by Whitesides and coworkers, a substrate such as a silicon (SiO_2_) wafer is brought in contact with a polydimethylsiloxane (PDMS)-based elastomeric template featuring recessed pattern of micrometer length scale. The PDMS, being soft, forms a tight contact with the substrate. A mixture of monomers, **1** (2 kDa) and **2** (2 kDa) is brought in contact with the substrate-template assembly to fill the relief features through a capillary action. The amine-epoxy polymerization process then solidifies the fluid and the mold can be peeled away to reveal the complementary poly(β-hydroxyl amine) micro-pattern on the substrate. As can be seen in Figure 8, interconnected channels in the template produced a mesh hydrogel structure with high fidelity of the features. 

## 4. Conclusions

A number of polyethylene glycol-based hydrophilic amine and epoxy precursors are available commercially. The epoxy groups in these precursors can be subjected to a ring-opening reaction by the amine nucleophiles. This reaction is catalyst-free and can be carried out in water. Therefore, a post-synthesis purification step is not required and the gels can be used as produced. The gelation, on the other hand, is a simple mixing of the precursors in an open-air and bench-top condition. The molecular weight of the precursors controls their gelation behavior and properties of the resulting hydrogels. Smaller precursors require a shorter gelation time and produce stiffer materials. Longer precursors require longer gelation time and higher temperatures but produce softer materials with high water uptake capacity. A balance is offered by 2 kDa precursors that offer good mechanical and water uptake properties. After the gelation reaction, the nitrogen atom of the amine group in the hydrogel scaffold can be alkylated through ring-opening reaction with the cyclic sultone to obtain zwitterionic materials. Finally, micromolding in capillaries is shown to be an applicable method for patterning amine-epoxy gel microstructures. 

## Figures and Tables

**Table 1:** Variation in the precursor chain length in 1 and 2.

Entry	1/2	1/2 (g)	Molar Ratio (1:2)	Solvent (mL)	Gel Formation (R.T)	Gel Formation (70 °C)	Rotational Rheometer (70 °C)	Young′s Modulus (kPa)
1	500/150	0.1/0.0015	2:1	H_2_O (0.115)	2.5–3 h	10 min	4 min	1575
2	500/2k	0.05/0.1	2:1	H_2_O (0.15)	2.5–3 h	20–30 min	13 min	659
3	500/10k	0.01/0.1	2:1	H_2_O (0.11)	−12 h	1–2 h	3 h	96
4	1k/150	0.1/0.0075	2:1	H_2_O (0.1075)	3–4 h	10–20 min	10 min	663
5	1k/2k	0.05/0.05	2:1	H_2_O (0.1)	5–6 h	20–30 min	18 min	571
6	1k/10k	0.02/0.1	2:1	H_2_O (0.12)	−12 h	1–2 h	3 h	117
7	2k/150	0.1/0.00375	2:1	H_2_O (0.1038)	6–7 h	20–30 min	20 min	339
8	2k/2k	0.1/0.05	2:1	H_2_O (0.15)	−12 h	40–50 min	33 min	601
9	2k/10k	0.04/0.1	2:1	H_2_O (0.14)	−12 h	3 h	3 h	260
10	6k/150	0.12/0.0015	2:1	H_2_O (0.1215)	−12 h	3 h	- ^a^	-
11	6k/2k	0.1/0.016	2:1	H_2_O (0.116)	> -	3–4 h	- ^a^	-
12	6k/10k	0.05/0.041	2:1	H_2_O (0.091)	> -	−12 h	- ^a^	-

^a^ a sticky semi-solid was obtained.

**Table 2:** Variation in the molar ratio of 1 (*M*_n_ = 500 g/mol) and 2 (*M*_n_ = 150 g/mol) in gelation studies.

Entry	1/2	1/2 (g)	Molar Ratio (1:2)	Solvent (mL)	Gel Formation (R.T)	Gel Formation (70 °C)	Rotational Rheometer (R.T)	Young′s Modulus (kPa)
1	500/150	1/0.0838	2:0.56	H_2_O (1.0838)	6–7 h	20–30 min	374 min	32
2	500/150	1/0.0915	2:0.61	H_2_O (1.0915)	3–4 h	10–20 min	225 min	135
3	500/150	1/0.0992	2:0.66	H_2_O (1.0992)	2–3 h	10–20 min	179 min	166
4	500/150	1/0.1068	2:0.71	H_2_O (1.1068)	2–2.5 h	10 min	154 min	328
5	500/150	1/0.1500	2:1	H_2_O (1.15)	1.5–2 h	10 min	114 min	1575

## Supplementary Materials

The following are available online at https://www.mdpi.com/2073-4360/11/9/1491/s1, Figure S1: Real-time rheological study of gelation with different precursors. Figure S2: Scanning electron micrographs (SEM) of various hydrogel samples. Figure S3: ^1^H NMR of **5** in deuterated H_2_O. Residual solvent signal is shown with an asterisk. Figure S4: ^1^H NMR of **9** in deuterated H_2_O. Residual solvent signals are shown with an asterisk. Figure S5: Fluorescence emission intensity of as-made and zwitterionic hydrogels after exposure to the protein BSA for a period of 24 h.

## References

[1] Muzammil E.M., Khan A., Stuparu M.C. (2017). Post-polymerization modification reactions of poly (glycidyl methacrylate)s. RSC Adv..

[2] Stuparu M.C., Khan A. (2016). Thiol-epoxy “click” chemistry: Application in preparation and postpolymerization modification of polymers. J. Polym. Sci. A.

[3] Chang H.-T., Fréchet J.M. (1999). Proton-transfer polymerization: A new approach to hyperbranched polymers. J. Am. Chem. Soc..

[4] Emrick T., Chang H.-T., Frechet J.M. (1999). An A2+ B3 approach to hyperbranched aliphatic polyethers containing chain end epoxy substituents. Macromolecules.

[5] Gong C., Fréchet J.M. (2000). Proton transfer polymerization in the preparation of hyperbranched polyesters with epoxide chain-ends and internal hydroxyl functionalities. Macromolecules.

[6] Hecht S., Emrick T., Fréchet J.M. (2000). Hyperbranched porphyrins—A rapid synthetic approach to multiporphyrin macromolecules. Chem. Commun..

[7] Emrick T., Chang H.-T., Fréchet J.M., Woods J., Baccei L. (2000). Hyperbranched aromatic epoxies in the design of adhesive materials. Polym. Bull..

[8] Lee B.F., Kade M.J., Chute J.A., Gupta N., Campos L.M., Fredrickson G.H., Kramer E.J., Lynd N.A., Hawker C.J. (2011). Poly (allyl glycidyl ether)-A versatile and functional polyether platform. J. Polym. Sci. A.

[9] Herzberger J., Niederer K., Pohlit H., Seiwert J., Worm M., Wurm F.R., Frey H. (2015). Polymerization of ethylene oxide, propylene oxide, and other alkylene oxides: Synthesis, novel polymer architectures, and bioconjugation. Chem. Rev..

[10] Brändle A., Khan A. (2012). Thiol–epoxy ‘click’polymerization: Efficient construction of reactive and functional polymers. Polym. Chem..

[11] Cengiz N., Rao J., Sanyal A., Khan A. (2013). Designing functionalizable hydrogels through thiol–epoxy coupling chemistry. Chem. Commun..

[12] Gadwal I., Binder S., Stuparu M.C., Khan A. (2014). Dual-reactive hyperbranched polymer synthesis through proton transfer polymerization of thiol and epoxide groups. Macromolecules.

[13] Binder S., Gadwal I., Bielmann A., Khan A. (2014). Thiol-epoxy polymerization via an AB monomer: Synthetic access to high molecular weight poly (β-hydroxythio-ether) s. J. Polym. Sci. A.

[14] Hwang J., Choe Y., Bang J., Khan A. (2017). Scalable ambient synthesis of water-soluble poly (β-hydroxythio-ether) s. J. Polym. Sci. A.

[15] Hwang J., Lee D.G., Yeo H., Rao J., Zhu Z., Shin J., Jeong K., Kim S., Jung H.W., Khan A. (2018). Proton transfer hydrogels: Versatility and applications. J. Am. Chem. Soc..

[16] Kolb H.C., Finn M., Sharpless K.B. (2001). Click chemistry: Diverse chemical function from a few good reactions. Angew. Chem..

[17] Ren Y., Jiang X., Yin J. (2009). Poly (ether tert-amine): A novel family of multiresponsive polymer. J. Polym. Sci. A.

[18] Saha A., De S., Stuparu M.C., Khan A. (2012). Facile and general preparation of multifunctional main-chain cationic polymers through application of robust, efficient, and orthogonal click chemistries. J. Am. Chem. Soc..

[19] Si G., Elzes M., Engbersen J., Paulusse J. (2018). Modular synthesis of bioreducible gene vectors through polyaddition of N, N′-dimethylcystamine and diglycidyl ethers. Polymers.

[20] Li D., Bu Y., Zhang L., Wang X., Yang Y., Zhuang Y., Yang F., Shen H., Wu D. (2015). Facile construction of pH-and redox-responsive micelles from a biodegradable poly (β-hydroxyl amine) for drug delivery. Biomacromolecules.

[21] Sun G., Liu J., Wang X., Li M., Cui X., Zhang L., Wu D., Tang P. (2019). Fabrication of dual-sensitive poly (β-hydroxyl amine) micelles for controlled drug delivery. Eur. Polym. J..

[22] Su Z., Jiang X. (2016). Multi-stimuli responsive amine-containing polyethers: Novel building blocks for smart assemblies. Polymer.

[23] Yu J., Su Z., Xu H., Ma X., Yin J., Jiang X. (2015). One-pot approach to synthesize hyperbranched poly (thiol–ether amine)(hPtEA) through sequential “thiol–ene” and “epoxy–amine” click reactions. Polym. Chem..

[24] Xu L.Q., Pranantyo D., Neoh K.-G., Kang E.-T., Teo S.L.-M., Fu G.D. (2016). Synthesis of catechol and zwitterion-bifunctionalized poly (ethylene glycol) for the construction of antifouling surfaces. Polym. Chem..

[25] Drury J.L., Mooney D.J. (2003). Hydrogels for tissue engineering: Scaffold design variables and applications. Biomaterials.

[26] Hoare T.R., Kohane D.S. (2008). Hydrogels in drug delivery: Progress and challenges. Polymer.

[27] Grinstaff M.W. (2008). Dendritic macromers for hydrogel formation: Tailored materials for ophthalmic, orthopedic, and biotech applications. J. Polym. Sci. A.

[28] Slaughter B.V., Khurshid S.S., Fisher O.Z., Khademhosseini A., Peppas N.A. (2009). Hydrogels in regenerative medicine. Adv. Mater..

[29] Shoichet M.S. (2009). Polymer scaffolds for biomaterials applications. Macromolecules.

[30] DeForest C.A., Anseth K.S. (2012). Advances in bioactive hydrogels to probe and direct cell fate. Annu. Rev. Chem. Biomol. Eng..

[31] Malkoch M., Vestberg R., Gupta N., Mespouille L., Dubois P., Mason A.F., Hedrick J.L., Liao Q., Frank C.W., Kingsbury K. (2006). Synthesis of well-defined hydrogel networks using click chemistry. Chem. Commun..

[32] Yigit S., Sanyal R., Sanyal A. (2011). Fabrication and functionalization of hydrogels through “click” chemistry. Chem. Asian J..

[33] Nimmo C.M., Shoichet M.S. (2011). Regenerative biomaterials that “click”: Simple, aqueous-based protocols for hydrogel synthesis, surface immobilization, and 3D patterning. Bioconjugate Chem..

[34] Azagarsamy M.A., Anseth K.S. (2012). Bioorthogonal Click Chemistry: An Indispensable Tool to Create Multifaceted Cell Culture Scaffolds.

[35] Cengiz N., Gevrek T., Sanyal R., Sanyal A. (2017). Orthogonal thiol–ene ‘click’reactions: A powerful combination for fabrication and functionalization of patterned hydrogels. Chem. Commun..

[36] Gevrek T.N., Cosar M., Aydin D., Kaga E., Arslan M., Sanyal R., Sanyal A. (2018). Facile fabrication of a modular “catch and release” hydrogel interface: Harnessing thiol–disulfide exchange for reversible protein capture and cell attachment. ACS Appl. Mater. Interfaces.

[37] Arslan M., Gevrek T.N., Sanyal R., Sanyal A. (2015). Fabrication of poly (ethylene glycol)-based cyclodextrin containing hydrogels via thiol-ene click reaction. Eur. Polym. J..

[38] Park E.J., Gevrek T.N., Sanyal R., Sanyal A. (2014). Indispensable platforms for bioimmobilization: Maleimide-based thiol reactive hydrogels. Bioconjugate Chem..

[39] Beria L., Gevrek T.N., Erdog A., Sanyal R., Pasini D., Sanyal A. (2014). ‘Clickable’hydrogels for all: Facile fabrication and functionalization. Biomater. Sci..

[40] Kaga S., Yapar S., Gecici E.M., Sanyal R. (2015). Photopatternable “clickable” hydrogels: “Orthogonal” control over fabrication and functionalization. Macromolecules.

[41] Hamid Z.A., Blencowe A., Ozcelik B., Palmer J.A., Stevens G.W., Abberton K.M., Morrison W.A., Penington A.J., Qiao G.G. (2010). Epoxy-amine synthesised hydrogel scaffolds for soft-tissue engineering. Biomaterials.

[42] Ozcelik B., Brown K.D., Blencowe A., Daniell M., Stevens G.W., Qiao G.G. (2013). Ultrathin chitosan–poly (ethylene glycol) hydrogel films for corneal tissue engineering. Acta Biomater..

[43] Stevens L., Calvert P., Wallace G.G. (2013). Ionic-covalent entanglement hydrogels from gellan gum, carrageenan and an epoxy-amine. Soft Matter.

[44] Krüger A.J., Köhler J., Cichosz S., Rose J.C., Gehlen D.B., Haraszti T., Möller M., De Laporte L. (2018). A catalyst-free, temperature controlled gelation system for in-mold fabrication of microgels. Chem. Commun..

[45] Zhou C., Truong V.X., Qu Y., Lithgow T., Fu G., Forsythe J.S. (2016). Antibacterial poly (ethylene glycol) hydrogels from combined epoxy-amine and thiol-ene click reaction. J. Polym. Sci. A.

[46] Liu Z., Zhang C., Xu H., Ma X., Shi Z., Yin J. (2017). A Facile Method Synthesizing Hydrogel Using Hybranched Polyether Amine (hPEA) as Coinitiator and Crosslinker. Macromol. Chem. Phys..

[47] Zhang C., Liu Z., Zhang X., Shi Z., Xu H., Ma X., Yin J., Tian M. (2017). Polyetheramine (PEA): A versatile platform to tailor the properties of hydrogels via H-bonding interactions. Polym. Chem..

[48] Bakarich S.E., Balding P., Gorkin III R., Spinks G.M. (2014). Printed ionic-covalent entanglement hydrogels from carrageenan and an epoxy amine. RSC Adv..

[49] Ding J., Zhou C., Li K., Zhang A., Yao F., Xu L., Fu G. (2016). Preparation of well-defined fibrous hydrogels via electrospinning and in situ “click chemistry”. RSC Adv..

[50] Qian S., Zhou C., Xu L., Yao F., Cen L., Fu G. (2014). High strength biocompatible PEG single-network hydrogels. RSC Adv..

[51] Liu S., Oderinde O., Hussain I., Yao F., Fu G. (2018). Dual ionic cross-linked double network hydrogel with self-healing, conductive, and force sensitive properties. Polymer.

[52] Lin C.-C., Anseth K.S. (2009). PEG hydrogels for the controlled release of biomolecules in regenerative medicine. Pharm. Res..

[53] Zhu J. (2010). Bioactive modification of poly (ethylene glycol) hydrogels for tissue engineering. Biomaterials.

[54] Bakaic E., Smeets N.M., Hoare T. (2015). Injectable hydrogels based on poly (ethylene glycol) and derivatives as functional biomaterials. RSC Adv..

[55] Kim E., Xia Y., Whitesides G.M. (1995). Polymer microstructures formed by moulding in capillaries. Nature.

