# Supplementary material for: A Modular and Practical Synthesis of Zwitterionic Hydrogels through Sequential Amine-Epoxy “Click” Chemistry and N-Alkylation Reaction
Source: Polymers (Basel). 2019 Sep 12;11(9):1491. doi: 10.3390/polym11091491 (PMC6780745; doi:10.3390/polym11091491)
Supplement: Supplementary file 1 [file polymers-11-01491-s001.pdf]

A modular and practical synthesis of zwitterionic hydrogels through sequential amine-epoxy 'click' chemistry and N-alkylation reaction

Junki Oh, Kevin Injoe Jung, Hyun Wook Jung, and Anzar Khan*

E-mail: anzar@korea.ac.kr

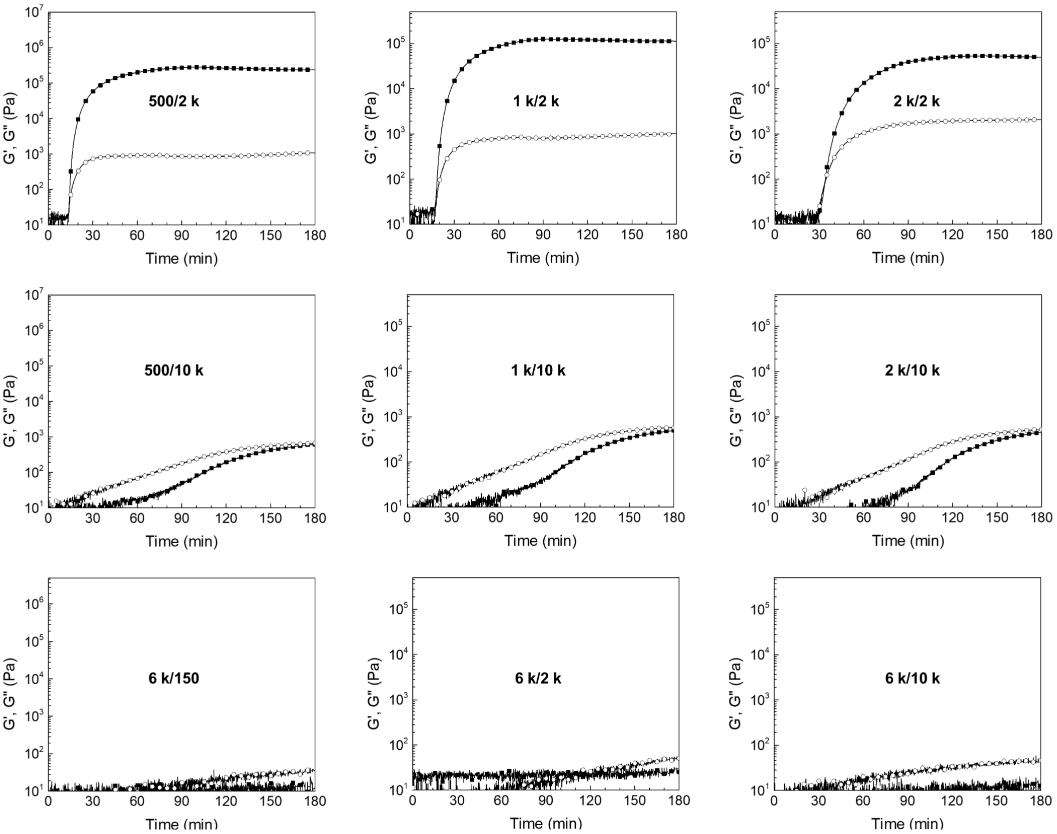

Figure S1. Real-time rheological study of gelation with different precursors.

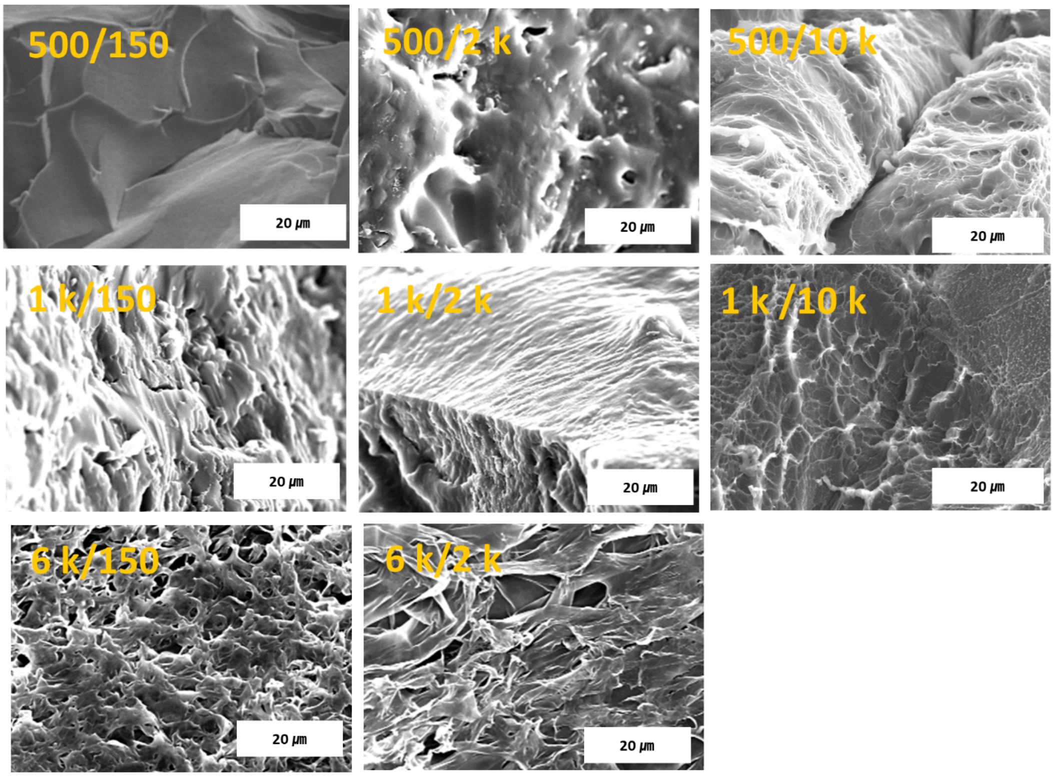

Figure S2. Scanning electron micrographs (SEM) of various hydrogel samples.

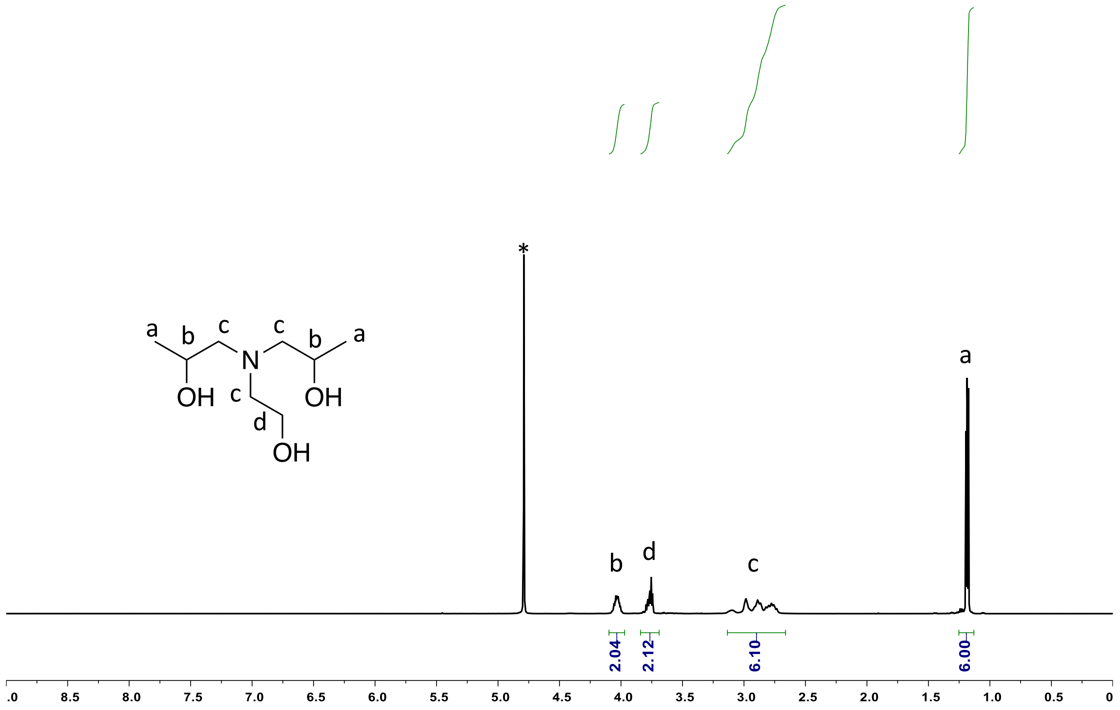

Figure S3. ^{1}H NMR of **5** in deuterated H_2O. Residual solvent signal is shown with an asterisk.

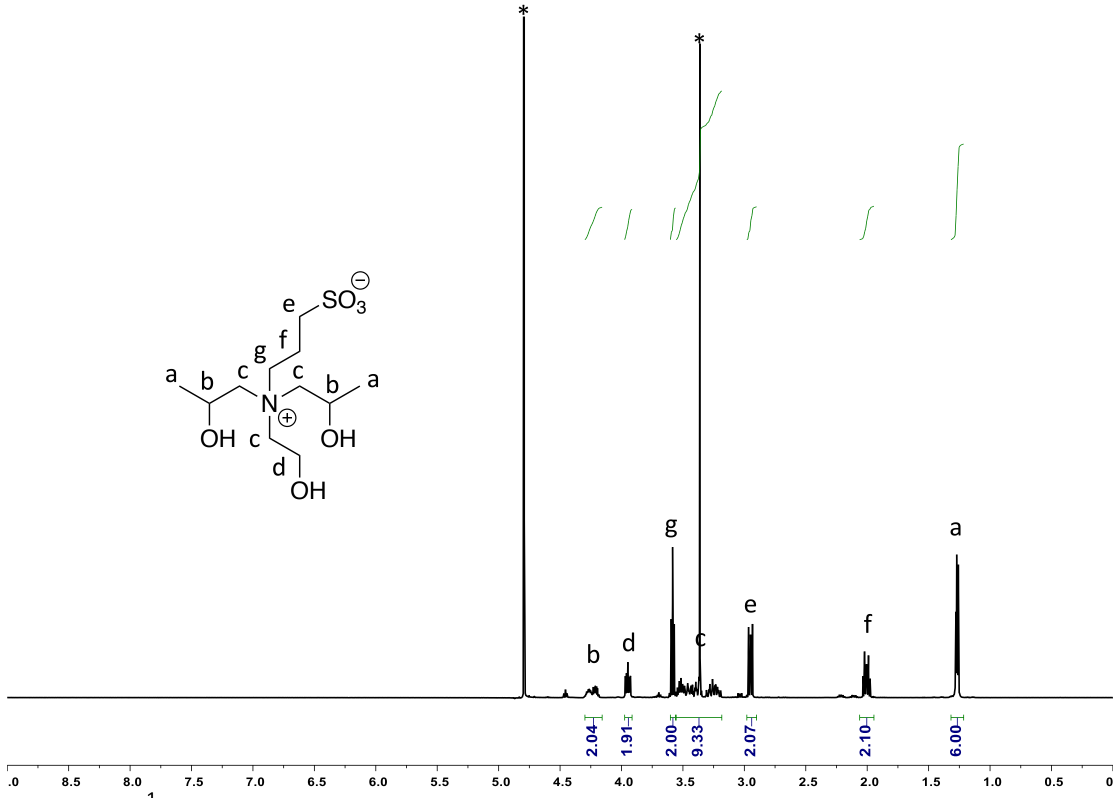

Figure S4. ^{1}H NMR of **9** in deuterated H_2O. Residual solvent signals are shown with an asterisk.

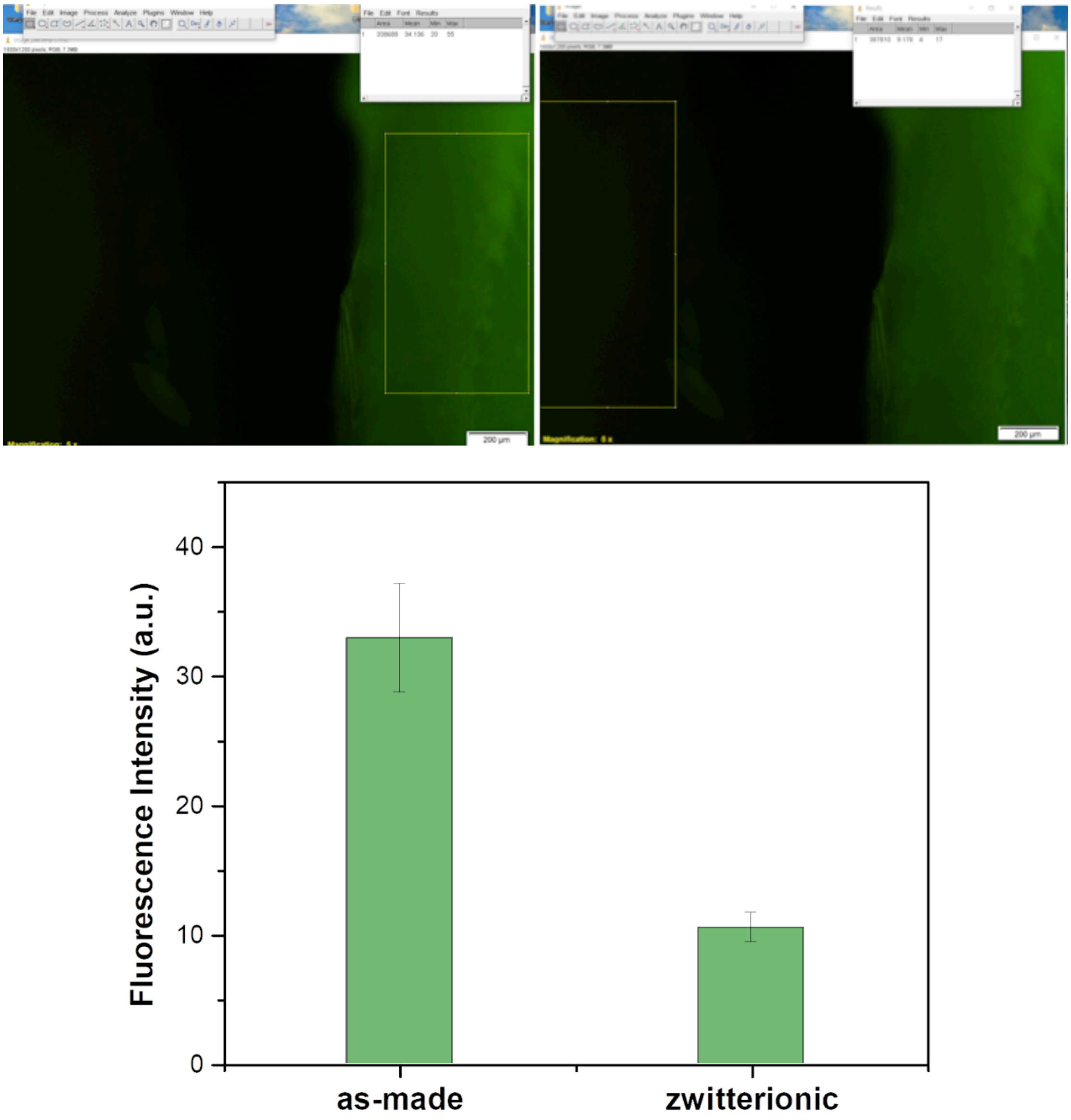

Figure S5. Fluorescence emission intensity of as-made and zwitterionic hydrogels after exposure to the protein BSA for a period of 24 h. The data is obtained from the top unmodified (left) and modified (right) gel surfaces.
